# Reduced deformability of parasitized red blood cells as a biomarker for anti-malarial drug efficacy

**DOI:** 10.1186/s12936-015-0957-z

**Published:** 2015-10-31

**Authors:** Xiaoyan Deng, Simon P. Duffy, Marie-Eve Myrand-Lapierre, Kerryn Matthews, Aline Teresa Santoso, Yi-Ling Du, Katherine S. Ryan, Hongshen Ma

**Affiliations:** Department of Mechanical Engineering, University of British Columbia, 2054-6250 Applied Science Lane, Vancouver, BC V6T 1Z4 Canada; Department of Chemistry, University of British Columbia, Vancouver, BC Canada; Department of Urologic Science, University of British Columbia, Vancouver, BC Canada; Vancouver Prostate Centre, Vancouver General Hospital, Vancouver, BC Canada

**Keywords:** Cell deformability, Malaria, *Plasmodium falciparum*, Drug screening, Biomarkers

## Abstract

**Background:**

Malaria remains a challenging and fatal infectious disease in developing nations and the urgency for the development of new drugs is even greater due to the rapid spread of anti-malarial drug resistance. While numerous parasite genetic, protein and metabolite biomarkers have been proposed for testing emerging anti-malarial compounds, they do not universally correspond with drug efficacy. The biophysical character of parasitized cells is a compelling alternative to these conventional biomarkers because parasitized erythrocytes become specifically rigidified and this effect is potentiated by anti-malarial compounds, such as chloroquine and artesunate. This biophysical biomarker is particularly relevant because of the mechanistic link between cell deformability and enhanced splenic clearance of parasitized erythrocytes.

**Methods:**

Recently a microfluidic mechanism, called the multiplexed fluidic plunger that provides sensitive and rapid measurement of single red blood cell deformability was developed. Here it was systematically used to evaluate the deformability changes of late-stage trophozoite-infected red blood cells (iRBCs) after treatment with established clinical and pre-clinical anti-malarial compounds.

**Results:**

It was found that rapid and specific iRBC rigidification was a universal outcome of all but one of these drug treatments. The greatest change in iRBC rigidity was observed for (+)-SJ733 and NITD246 spiroindolone compounds, which target the *Plasmodium falciparum* cation-transporting ATPase ATP4. As a proof-of-principle, compounds of the bisindole alkaloid class were screened, where cladoniamide A was identified based on rigidification of iRBCs and was found to have previously unreported anti-malarial activity with an IC_50_ lower than chloroquine.

**Conclusion:**

These results demonstrate that rigidification of iRBCs may be used as a biomarker for anti-malarial drug efficacy, as well as for new drug screening. The novel anti-malarial properties of cladoniamide A were revealed in a proof-of-principle drug screen.

**Electronic supplementary material:**

The online version of this article (doi:10.1186/s12936-015-0957-z) contains supplementary material, which is available to authorized users.

## Background

Malaria remains one of the greatest threats to human health. In 2013, there were an estimated 198 million cases resulting in 584,000 deaths [1], of which 90 % occurred in children under the age of five [2]. A number of effective anti-malarial drugs and derivatives have been developed. However, the emergence of drug-resistance [3] has prompted the urgent development of new anti-malarial compounds as well as protocols to screen these compounds. Existing methods to screen for anti-malarial activity are almost entirely based on parasite survival during the intra-erythrocytic stage and is typically assessed by microscopy, ELISA-based parasite antigen detection, fluorescent DNA-binding dyes, flow cytometry, and PCR of parasite-specific genes [4]. However, the measurement of parasite survival provides limited insight into the effectiveness of the candidate drug in vivo, therefore, requiring a large sub-set of compounds to be subsequently evaluated using *Plasmodium berghei* in murine infection models, using a malarial species different from those that infect humans. Subsequent human clinical trials are costly and are complicated by issues such as toxicity, bioavailability, patient physiology, and side-effects, resulting in extremely low success rates, even for compounds that showed high efficacy in vitro [5]. Owing to the poor success of existing screening methods, there is a need for in vitro screens that align with a common mode of action for anti-malarial compounds in vivo. This type of in vitro method would significantly reduce the burden of animal and human drug trials.

Specific reduced deformability of *Plasmodium falciparum* parasitized red blood cells (iRBCs) has been well documented. This biophysical change has been attributed to oxidative stress induced by the parasites’ metabolism of haemoglobin [6], remodelling of the iRBC cytoskeleton by parasite-derived proteins [7], as well as the presence of the enlarged digestive vacuole in the late stages of infection [8, 9]. Interestingly, this rigidification of iRBCs enhances their accumulation in micro-anatomical zones and the microcirculation of the spleen, and contributes to their splenic elimination [10]. Furthermore, anti-malarial drugs are likely to exaggerate iRBCs’ rigidification and subsequent splenic clearance, by preventing haemoglobin detoxification or increasing intracellular oxidation [11, 12]. Together, these observations suggest that enhanced splenic clearance of iRBCs due to their rigidification might contribute to the mode of action of anti-malarial compounds and would therefore be an important, clinically relevant biomarker for drug efficacy.

Previous studies have reported infection-induced rigidification of iRBCs following exposure to chloroquine or artesunate [13, 14]. However, parasitized cells may represent a small fraction of the erythrocyte population and this effect may not be detectable by conventional inference of average RBC deformability, based on the viscous properties of blood [15]. The microfluidic multiplexed Fluidic Plunger (MFP) overcomes this limitation by high-throughput measurement of single-cell deformation through microstructures that mimic capillaries in the microvasculature.

The current study aimed to determine whether changes in iRBC deformability correlate with anti-malarial efficacy. It was observed that iRBC rigidification was a common outcome of the anti-malarial drugs tested. Furthermore, *Pf*ATP4 inhibitors that induce rapid clearance of parasitized cells induced the greatest magnitude of iRBC deformability. Finally, in a small screen of bisindole alkaloid compounds, it was shown that changes in iRBC deformability could predict the previously unreported anti-malarial properties of cladoniamide A.

## Methods

### Cultivation of *Plasmodium falciparum* 3D7

Type A+ blood was collected from healthy donors with written informed consent and approval from the UBC Office of Research Ethics (Network Centre for Applied Development, Canadian Blood Services). Washed erythrocytes were then used to culture *P. falciparum* parasites (strain 3D7) (Network Centre for Applied Development, Canadian Blood Services) at 5 % haematocrit in culture media, consisting of RPMI-1640 medium (Invitrogen, CA, USA) containing 25 mM HEPES (Sigma-Aldrich, St Louis, MO, USA), 0.5 % AlbuMAX I (w/v) (Invitrogen), 100 µM hypoxanthine (Sigma-Aldrich), 12.5 µg/ml gentamicin (Invitrogen) and 1.77 mM sodium bicarbonate (Sigma-Aldrich) at pH 7.4. Cultures were grown in standard 90 mm Petri dishes (Corning Life Science, Tewksbury, MA, USA) at a final volume of 10 ml and were kept in a hypoxic incubator (Caron, Marietta, USA) with a standard gas environment of 5 % CO_2_, 1 % O_2_, and 94 % N_2_ at 37 °C.

### Purification of iRBCs and subsequent drug treatment

A magnetic purification stand was fabricated based on the design by Kim [16] with some modifications to fit super magnets. LS columns (Miltenyi Biotec, Bergisch Gladbach, Germany) were employed in the positive selection of haemozoin-containing iRBCs (late-stage trophozoite-iRBCs) by loading 30 ml of parasite culture at 2 % haematocrit (v/v) and 5–15 % parasitaemia. Washing media was prepared that was identical to culture media, with the exception of sodium bicarbonate. The columns were washed once with 5 ml washing media and the captured cells were eluted in another 5 ml washing media by centrifugation at 350×*g* for 5 min with no brakes. Enrichment purity of late-stage trophozoite-iRBCs was determined Giemsa-staining of an aliquot (Sigma-Aldrich). The remaining cells were suspended in culture media and acclimatized for 30 min within a hypoxic chamber, prior to incubation with drugs at specified concentrations and incubation times. Anti-malarial compounds were diluted in DMSO to a normalized concentration four times greater than their established EC50 [17–20], as indicated in Table 1. The EC_50_ for each candidate anti-malarial drug was not known a priori, so they were each tested at a concentration of 1 µM. RBCs from healthy donors were evaluated, incubated with 0.01 % DMSO or ddH_2_O, as well as each compound to assess whether the observed effect was specific for iRBCs. RBC deformability was determined by multiplexed fluidic plunger and normalized to the control DMSO-treated RBCs.

**Table 1:** Individual concentrations and deformability values that were normalized to DMSO or ddH_2_O control for respective anti-malarial drug treatments

Drug name	Concentration (≥4 × EC_50_)	Median normalized deformability	% Median increase in deformability	P value
Chloroquine	1 µM	1.41	41	<0.0001
Mefloquine	1 µM	1.22	22	0.0002
Pyrimethamine	20 nM	1.80	80	<0.0001
Proguanil	100 µM	1.58	58	0.0052
Artesunate	10 nM	1.29	29	0.0022
Artemether	8 nM	1.95	95	<0.0001
Dihydroartemisinin	20 nM	2.11	111	<0.0001
Tetracycline	100 µM	0.93	−7	ns
Atovaquone	250 nM	1.46	46	0.0001
(+)-SJ733	1.08 µM	5.54	454	<0.0001
(−)-SJ733	1.08 µM	0.90	−10	ns
NITD246	3.6 nM	3.34	234	<0.0001

### Microfluidic measurements of iRBC deformability

**Fig. 1:**
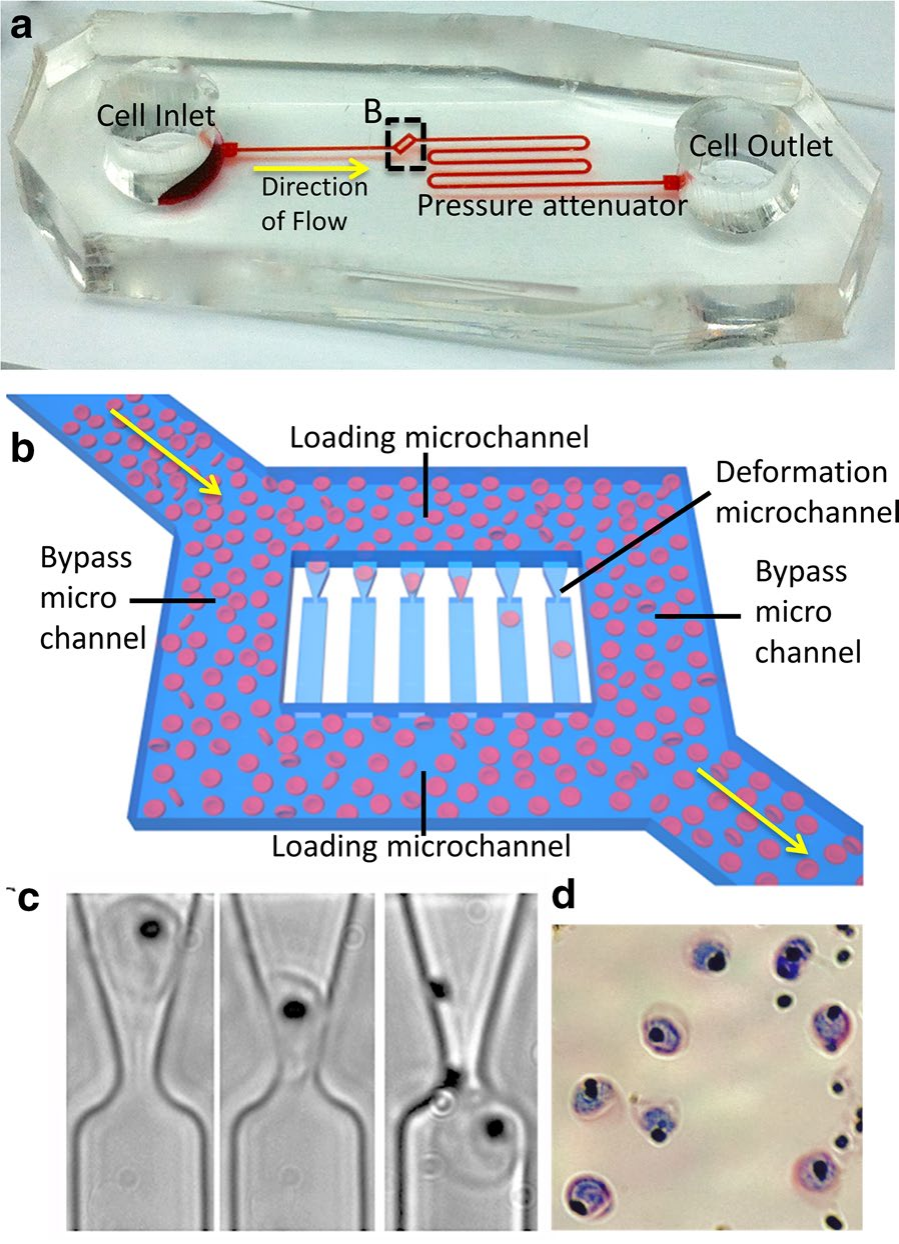
Overview of the multiplexed fluidic plunger mechanism used to measure trophozoite-infected red blood cells. **a** The microfluidic device measures ~40 mm by 20 mm and consists of a *cell* *inlet* and *outlet* reservoir, a long pressure attenuator and **b** deformability microarray, made up of a *rectangular* microchannel with 2 loading microchannels and 2 bypass microchannels surrounding 34 parallel deformation microchannels with a microconstriction width of ~2.5 µm and a height of ~3.7 µm. RBCs are loaded into the deformation microchannel under very low pressure. The pressure is then incrementally increased until the cell transits the microconstriction. **c** The pressure required for the trophozoite-infected RBC (RBCs with a *black* pigment) to transit the microconstriction is indicative of the cell’s deformability [21]. **d** Giemsa-stained blood smear confirming the pigmented iRBCs measured by the device are trophozoites

The deformability of iRBCs was measured using the previously developed multiplexed fluidic plunger microfluidic device (Fig. 1a, b) [21]. Briefly, the principle of the fluidic plunger mechanism involves infusing a single cell into a microchannel containing a constriction small enough to significantly deform the cell. When the cell is free flowing in the constriction, pressure applied across the microchannel is distributed along its length. Once the cell reaches the constriction, it forms a temporary seal against the constriction, causing the applied pressure to focus across the cell. Precisely controlling the applied pressure allows an adjustable stress to be applied remotely to a cell and constriction pair. For the experiments described in this study, a microchannel of 2.5 µm in width and 3.7 µm in thickness was used in order to constrain RBCs in a flat configuration parallel with the microchannel. A constriction aperture of 2.5 µm was used to constrain the RBCs and deformed them using pressure applied across the microchannel.

Multiplexing this process involves arranging multiple deformation microchannels in parallel, as well as designing supporting microchannels that ensure that a consistent pressure is applied to all microchannels independent of position and whether they are occupied by a RBC. These supporting microchannels consist of a rectangular detour around parallel deformation, along with longer microchannels leading to the sample inlet and outlet. The rectangular detour structure performs three key functions: (1) pressure applied across the deformation microchannels derives from pressure applied across the inlet and outlet and is reduced by a precise ratio that is set by the relative length of the inlet microchannel and part of the rectangular detour; (2) pressure applied across the deformation microchannels is independent of position in the deformation microchannel array by the symmetric fluidic structure of the rectangular detour; and, (3) pressure applied across the deformation microchannel is made independent of the number of cells being deformed by the relative hydrodynamic resistance of the deformation microchannels and the rectangular detour microchannel. Detailed technical design of the deformation microchannels and the rectangular detour are described in Myrand-Lapierre et al. [21] The device used for this study consists of 34 deformation microchannels in parallel with a rectangular detour microchannel of 25 µm in thickness and 250 µm in width.

### In vitro drug sensitivity test by the SYBR green I assay

In vitro parasitized erythrocyte cultures were maintained such that the proportion of ring-stage parasites never exceeded 70 % and a parasitaemia of 5–8 % was established prior to drug sensitivity assay. The cells were collected by centrifugation at 350×*g* for 5 min and re-suspended in RPMI culture media to a parasitaemia of 0.5 %, and the haematocrit was adjusted to 2 % with fresh unparasitized RBCs. Parasitized RBCs were transferred to a 96-well culture plate (Corning, NY, USA) at 200 μl/well of 0.5 % parasitized RBCs, at 2 % haematocrit. Each compound was added to the specified concentration (Table 1) and incubated in a hypoxic incubator at 37 °C (5 % O_2_, 5 % CO_2_, 90 % N_2_) for 24 h. Uninfected RBCs treated with 0.01 % DMSO (Sigma-Aldrich) and each test compound while iRBCs were also treated with 0.01 % DMSO alone, as controls. Following incubation, the plates were frozen and stored at −80 °C. Prior to assay, samples were thawed for 1 h at room temperature and mixed by pipetting. A total of 100 μl of each culture was mixed with 100 μl of 1× SYBR in 2× Lysis buffer [20 mM Tris; 0.008 % Saponin (w/v); 0.08 % Triton X-100 (w/v)] and transferred to a new 96-well plate. Fluorescence signal was determined in duplicate using a Biomek FX Multimode Detector (Beckman Coulter, Pasadena, CA, USA) at an excitation wavelength of 485 nm and an emission wavelength of 535 nm.

### Statistical analysis

Median RBC deformability and interquartile range (IQR) was calculated using Graphpad Prism v5. A Student *t* test was used to assess statistical significance between normally distributed RBC deformability profiles. The relationship between changes in RBC deformability and anti-malarial drug incubation time or concentration was evaluated by linear regression, while non-linear regression analysis was used to analyse the in vitro IC_50_ of new compounds on parasites.

## Results

### Multiplexed fluidic plunger detected time-dependent rigidification of chloroquine-treated iRBCs

**Fig. 2:**
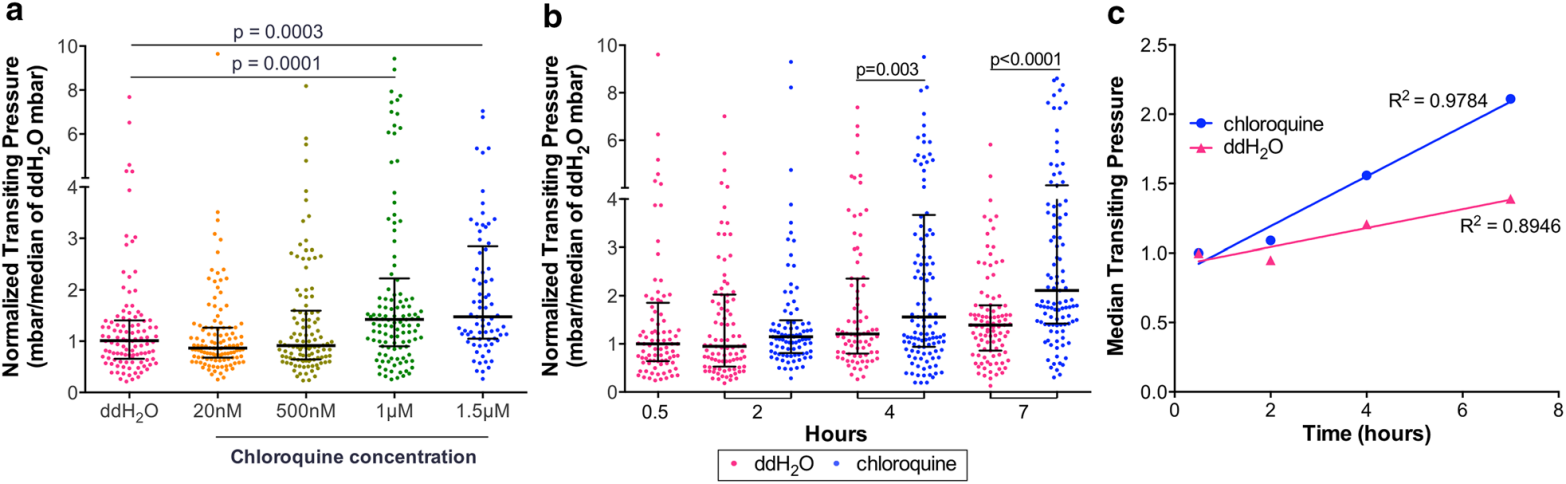
Chloroquine exposure significantly decreases the deformability of trophozoites-infected red blood cells in a dose- and time-dependent manner. **a** Purified trophozoites were incubated with a range of chloroquine concentrations for 4 h. Each data point represents the transit pressure for one trophozoite iRBC and a significant increase in transit pressure was observed a 1 and 1.5 µM. **b** Purified trophozoites were incubated with 1 µM chloroquine for at 2, 4 or 7 h. Treated and untreated iRBCs showed time-dependent loss of deformability, with a significant increase in transit pressure by 4 h (p = 0.0026), compared to untreated iRBCs. **c** There is a positive correlation between the median transiting pressure of iRBCs incubated with (R^2^ = 0.9784) and without (R^2^ = 0.8946) chloroquine treatment and time of incubation

Alteration in RBC deformability after *P. falciparum* infection and anti-malarial drug treatment was assessed using the MFP device [21], which measures the deformability of single RBCs based on the pressure required to transit a funnel-shaped microconstriction (Fig. 1). To evaluate the sensitivity of this system for measuring drug-induced changes in RBC deformability, the deformability of purified *P. falciparum* 3D7 iRBCs was determined over a range of chloroquine concentrations by measuring the median transit pressure through a microscale funnel (Fig. 2a). While chloroquine did not affect the median transit pressure for uninfected RBCs (Additional file 1: Figure S1), iRBCs required significantly greater transit pressure at 1 µM (p = 0.0002) and 1.5 µM (p = 0.0006) of chloroquine treatment, compared to untreated controls. Both untreated and chloroquine-treated iRBCs showed a time-dependent rigidification. However, following 4 h of incubation, the chloroquine-treated iRBCs were significantly more rigid than untreated iRBCs (Fig. 2b, p = 0.0026). Furthermore, there is a direct linear correlation between chloroquine incubation time and iRBC transit pressure (Fig. 2c, R^2^ = 0.9784).

### Measurement of iRBC deformability following treatment with a panel of conventional anti-malarial compounds

**Fig. 3:**
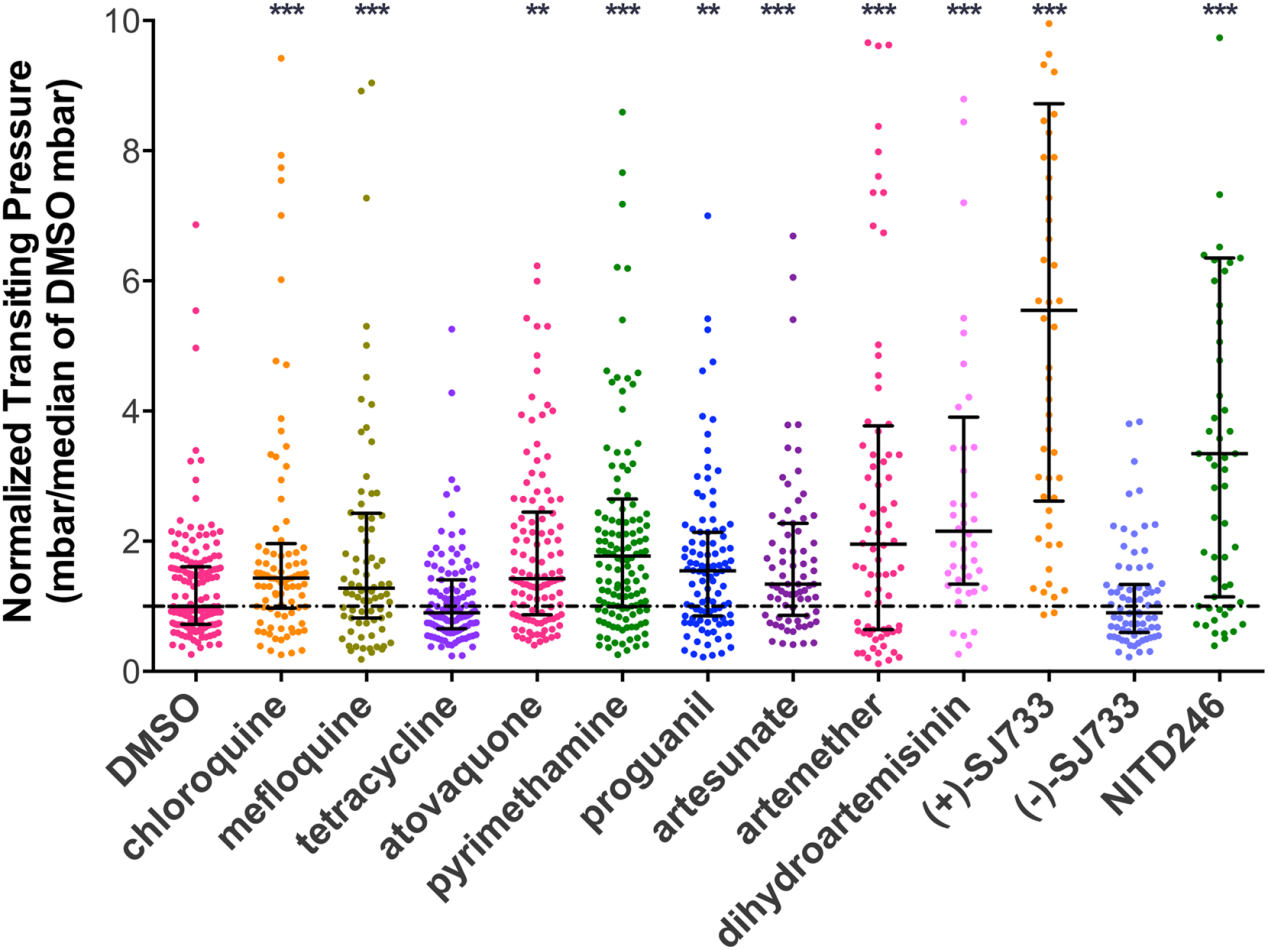
Anti-malarial drugs impair the deformability of trophozoite-infected red blood cells. The deformability of purified trophozoite-iRBCs was significantly diminished by treatment with all anti-malarial drugs tested, except tetracycline. Treatment with the novel spiroindolone NITD246 and DHIQ—(+) SJ733 showed the highest transiting pressure required for the iRBCs to squeeze through the microconstrictions of the device. **p < 0.001, ***p < 0.0001

Previous studies have reported that both chloroquine and artesunate treatment contribute to a specific rigidification of iRBCs [13, 14]. However, it was uncertain whether this was a common feature of anti-malarial compounds or whether this phenomenon was restricted to these specific drugs. To assess whether iRBC rigidification was a universal outcome of anti-malarial drug treatment, the effect of 12 established anti-malarial compounds on iRBC deformability were systematically assessed. Purified iRBCs were incubated with drugs at a specific concentration (≥4 × EC_50_) for 4h and alteration in the biophysical characteristics of the cell were assessed by MFP. None of these anti-malarial compounds significantly altered the deformability of uninfected RBCs (Additional file 2: Figure S2). Suspensions of iRBCs in 0.001 % DMSO and ddH_2_O were used as baseline controls for this experiment and there was no significant difference in median transit pressure between these control iRBCs. In contrast to untreated controls, a significant increase in median transit pressure was observed for all drug-treated iRBCs, except tetracycline and the (−)-SJ733 enantiomer that serves as a negative control with no anti-malarial activity (Fig. 3, p < 0.001).

It was observed that the different anti-malarial compounds had different effects on the deformability of iRBCs (Table 1). For example, while mefloquine and chloroquine are related compounds, mefloquine only reduced iRBC deformability by 22 %, while chloroquine induced 44 % reduction in iRBC deformability. Similarly, it was observed that each of the artemisinin derivatives induced significant rigidification of iRBCs and among them, dihydroartemisinin resulted in the least deformable iRBCs (111 %). The greatest rigidified iRBCs resulted from treatment with NITD 246 and the active (+)-SJ733 enantiomer, showing 228 and 469 % loss in deformability, respectively, relative to the control (Fig. 3; Table 1).

### Screen of bisindole alkaloid compounds for anti-malarial activity

**Fig. 4:**
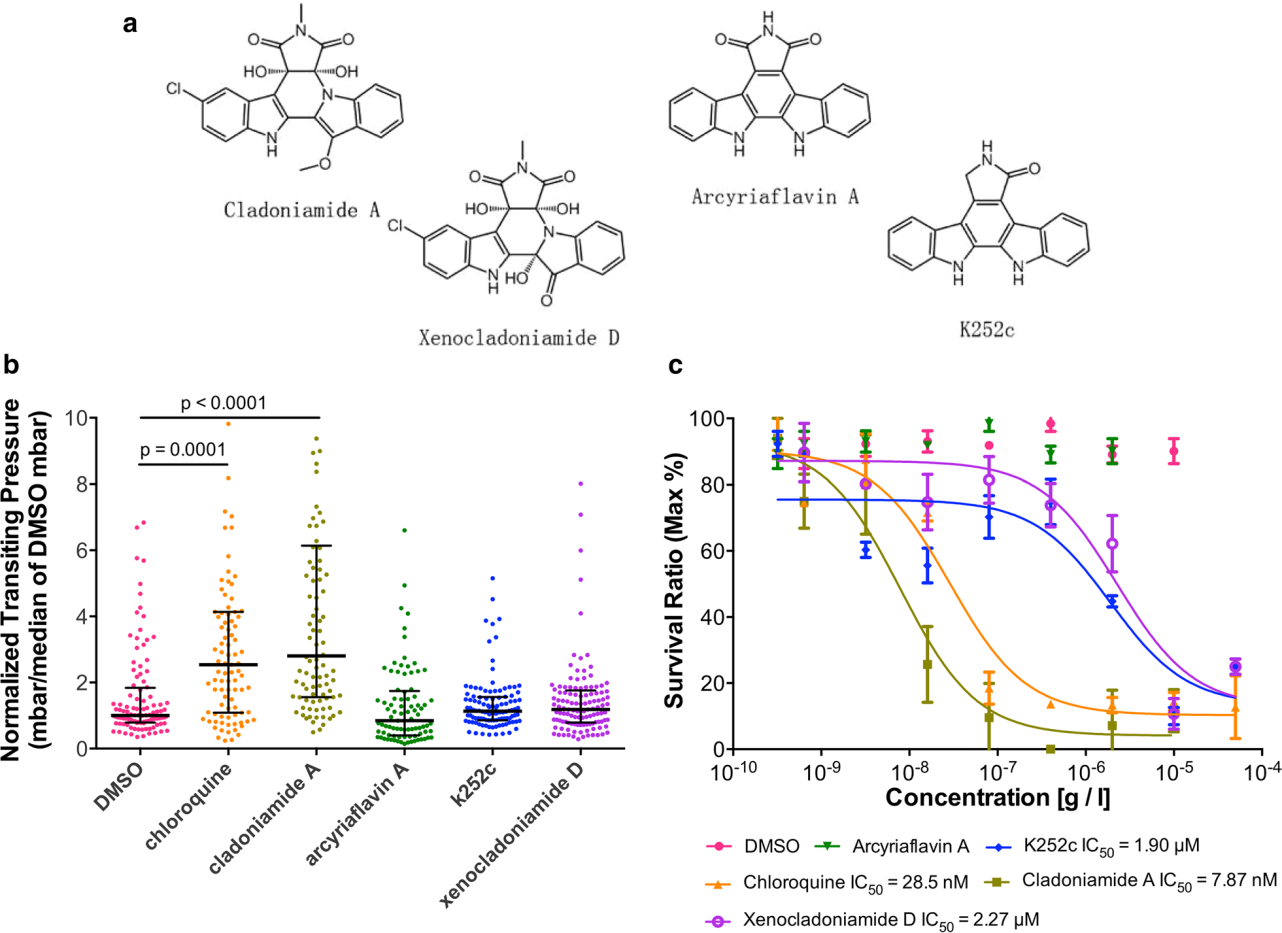
‘Mock’ drug screen shows the potential of cladoniamide A as an anti-malarial. **a** Chemical structures of the bisindoles investigated in this study. **b** With reference to chloroquine, Bisindole compounds (1 µM each) exert differential effects on iRBC deformability. Cladoniamide A (p < 0.0001) and chloroquine (p = 0.0001) significantly decreased iRBC deformability and K252c and xenocladoniamide D treatment resulted in non-significant reduction in deformability. **c** The in vitro sensitivity of cladoniamide compounds tested by the SYBR Green I assay. The IC_50_ was 9.041 nM for cladoniamide A, 28.46 nM for chloroquine, 1.899 µM for K252c, and 2.271 µM for xenocladoniamide D

Collectively, these data suggested that iRBC rigidification could be an alternative method for anti-malarial drugs screening. Therefore, as a proof-of-principle, a group of bisindole alkaloid compounds were tested, which derive biosynthetically from the oxidative dimerization of tryptophan [22–24] for their effects on iRBCs’ rigidification. Specifically, cladoniamide A, xenocladoniamide D, arcyriaflavin A, and K252c were examined and it was observed that (Fig. 4a) cladoniamide A imparted 17 % greater rigidification of iRBCs than did chloroquine (Fig. 4b, p < 0.0001). Xenocladoniamide D and K252c resulted in a slight non-significant rigidification of iRBC relative to the DMSO control and arcyriaflavin A had no effect. None of these compounds affected the deformability of uRBCs (Additional file 3: Figure S3).

To assess whether the specific induced iRBC rigidification observed for cladoniamide A correlated with anti-malarial activity, an in vitro SYBR-Green fluorescence assay was performed to measure the effect of each drug on parasitaemia and generated dose response curves to determine the IC_50_ for each compound. Consistent with the deformability analysis chloroquine (IC_50_ = 28 nM) and cladoniamide A (IC_50_ = 7.8 nM) displayed anti-malarial activity at a significantly lower dose than xenocladoniamide D (IC_50_ = 2.27 μM) and K252c (IC_50_ = 1.9 μM) (Fig. 4c). The anti-malarial activity of cladoniamide A has not been previously reported. However, it is interesting to note that cladoniamide A was recently reported to target a V-Type H^+^-ATPase in yeast [25]. In the *Plasmodium* parasite the V-type H^+^-ATPase maintains the intracellular pH and disruption of this transporter contributes to acidification of the parasite [25]. Another prominent anti-malarial drug target is *Pf*ATP4 which employs efflux to maintain a low parasite intracellular Na^+^ concentration in Na^+^-rich blood. However, it has been reported that malaria V-Type H^+^-ATPase activity is Na^+^-dependent, suggesting mechanistic link between these transporters and *Pf*ATP4 [26]. Together, these findings suggest that alterations in iRBC deformability correlate with anti-malarial activity. This rapid deformability-based screen was employed to identify the putative V-Type H^+^-ATPase inhibitor cladoniamide A as a compound with potent anti-malarial activity.

## Discussion

This study involved systematic assessment of the effect of established clinical and pre-clinical anti-malarial compounds on the deformability of parasitized RBCs. It was observed that decrease in cell deformability was a common outcome of nearly all established anti-malarial compounds tested. Furthermore, using specific reduced iRBC deformability as a biomarker for drug efficacy, cladoniamide A was identified as a candidate novel anti-malarial compound. Further investigation indicated that this compound has an IC_50_ that is lower than chloroquine.

While most of the anti-malarial compounds examined in this study corresponded with a decrease in iRBC deformability, they likely do so by distinct cellular mechanisms. The *Plasmodium* parasite metabolizes the haemoglobin of the host erythrocyte and generates toxic haem as a byproduct. Normally, the parasite detoxifies the host cell by biocrystallization of haem into haemozoin, but chloroquine inhibits this process and inhibits haem degradation to collectively contribute to the accumulation of haem within the host cell [27]. Intracellular haem contributes to oxidative stress in the iRBC, which is thought to be directly toxic to the parasite based on the observed sensitivity of *P. falciparum* to pro-oxidants [28], such as nitric oxide [29], hydrogen peroxide and superoxide [30]. Furthermore, oxidative stress contributes to reduced cell deformability by membrane lipid peroxidation [11]. Also, quinoline anti-malarial drugs, such as chloroquine and mefloquine, induce haem association with the cell membrane, which further impairs the integrity of the membrane [28]. The observation that mefloquine has a more subtle impact on iRBC deformability than chloroquine is consistent with observation that mefloquine is a less effective inhibitor of haem degradation [28]. In contrast to the quinolone drugs, the mechanism for artesunate anti-malarial activity is not well established. However, artemisinin derivatives, such as artesunate, arthemether and dihydroartemisinin, are thought to be rapidly hydrolyzed or demethylated to their active metabolite, dihydroartemisinin [31, 32], which is therapeutically active against malaria infection [33]. Artemisinins may also target sarco/endoplasmic reticulum Ca^2+^-ATPase, *P. falciparum* phosphatidylinositol-3-kinase and haemoglobin digestion [34]. These cellular processes stimulate reactive oxygen species (ROS) that oxidize membrane thiols and contribute to reduced cellular deformability [12].

NITD 246 and the active (+)-SJ733 enantiomer induced the greatest rigidification of iRBCs. Both active molecules belong to the novel anti-malarial spiroindolone family that target *Pf*ATP4, a P-type ATPase [20, 35, 36]. The uptake of important nutrition by invading parasites allows Na^+^ influx and K^+^ efflux at the same time [36]. *Pf*ATP4, a parasite plasma membrane protein, helps intra-erythrocytic parasites maintain a low cytosolic Na^+^ by extruding Na^+^ against an inward gradient in exchange for H^+^ entry [36]. NITD246 and (+)-SJ733 work as inhibitors of *Pf*ATP4 in nanomolar concentrations and perturb efflux of Na^+^ and coupled influx of H^+^ by *Pf*ATP4 into the parasites, which increases both intracellular pH and sodium concentrations, resulting in parasite swelling and death [20, 36] as well as a spherical morphology of the RBC [20]. The (−)-SJ733 enantiomer was unable to reduce the deformability of iRBCs, consistent with its low potency against malaria in vitro [20] and highlights the specificity of the anti-malarial property to (+)-SJ733.

As a proof-of-principle, a group of bisindole alkaloid compounds were screened. Specifically, cladoniamide A, xenocladoniamide D, arcyriaflavin A, and K252c were examined. Cladoniamide A is a nanomolar cytotoxic agent, thought to target vacuolar H^+^-ATPase [25], xenocladoniamide D is an inhibitor of the colon cancer cell line HCT-116 [23], arcyriaflavin A inhibits human cytomegalovirus replication in vitro, and K252c inhibits many protein kinases, such as protein kinase C [37]. None of these compounds has been previously reported to have anti-malarial properties and none of them affected the deformability of uRBCs. The observation that cladoniamide A induces a dramatic rigidification of iRBCs and that this corresponds with a potent anti-malarial effect serves to support the hypothesis that reduced iRBC deformability is indicative of anti-malarial drug efficacy.

## Conclusion

These results suggest that specific rigidification of iRBCs is a feature common to the anti-malarial compounds tested. Moreover, the propensity for less deformable cells to be retained within the interendothelial clefts of the spleen [10], suggest that changes in iRBC deformability may contribute to the mode of action of anti-malarial compounds. Support for this assertion can be drawn from studies where chloroquine-mediated changes in RBC deformability promote more rapid clearance of the rigid cells [14]. Furthermore, the observation that the spiroindolones NITD246 and (+)-SJ733 induced the greatest magnitude of iRBC rigidification is consistent with observations that the primary mode of action for these *Pf*ATP4 inhibitors is by host-mediated clearance [20]. The progressive rigidification of parasitized RBCs has previously been attributed to parasite-expressed factors as well as enhanced cell senescence and that rigid RBCs contribute to disease pathology through sequestration of these cells and obstruction of the microvasculature. However, observation that rigidification is a feature common to clinical ant-malarial compounds tested here, suggest that its impact is primarily associated with clearance of infected RBCs by adsorption within the inter-endothelial clefts of the spleen. Finally, a small-scale screen of bisindole alkaloids revealed that reduced RBC deformability could serve as a predictor of anti-malarial activity in a compound. Together, these data, as well as recently published data [38], suggest that reduced cell deformability can serve as a rapid and sensitive biomarker for anti-malarial drug efficacy. The ability to assess cell deformability using massively parallelized microfluidic systems without the need for expensive reagents, make such systems ideal for large-scale anti-malarial drug screens.

## Additional files

10.1186/s12936-015-0957-z Biophysical response of uninfected red blood cells to chloroquine. Neither (A) chloroquine concentration nor (B) incubation time significantly affected the deformability of uninfected RBCs.
10.1186/s12936-015-0957-z Biophysical response of uninfected red blood cells to anti-malarial compounds. The anti-malarial compounds tested in this study did not significantly affect the deformability of uninfected RBCs.
10.1186/s12936-015-0957-z Biophysical response of uninfected red blood cells to bisindole alkaloid compounds. Bisindole alkaloids tested did not significantly affect the deformability of uninfected RBCs.
