# Supplementary figures and images for: Reduced deformability of parasitized red blood cells as a biomarker for anti-malarial drug efficacy
Source: Malar J. 2015 Oct 31;14:428. doi: 10.1186/s12936-015-0957-z (PMC4628286; doi:10.1186/s12936-015-0957-z)

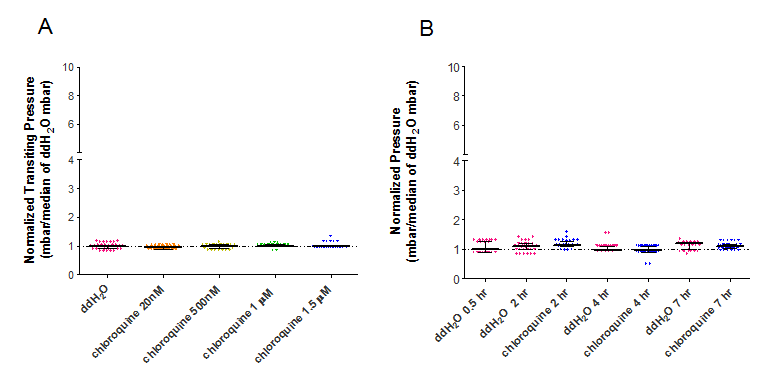

Supplement: Supplementary file 1 — 10.1186/s12936-015-0957-z Biophysical response of uninfected red blood cells to chloroquine. Neither (A) chloroquine concentration nor (B) incubation time significantly affected the deformability of uninfected RBCs. [file 12936_2015_957_MOESM1_ESM.tif]

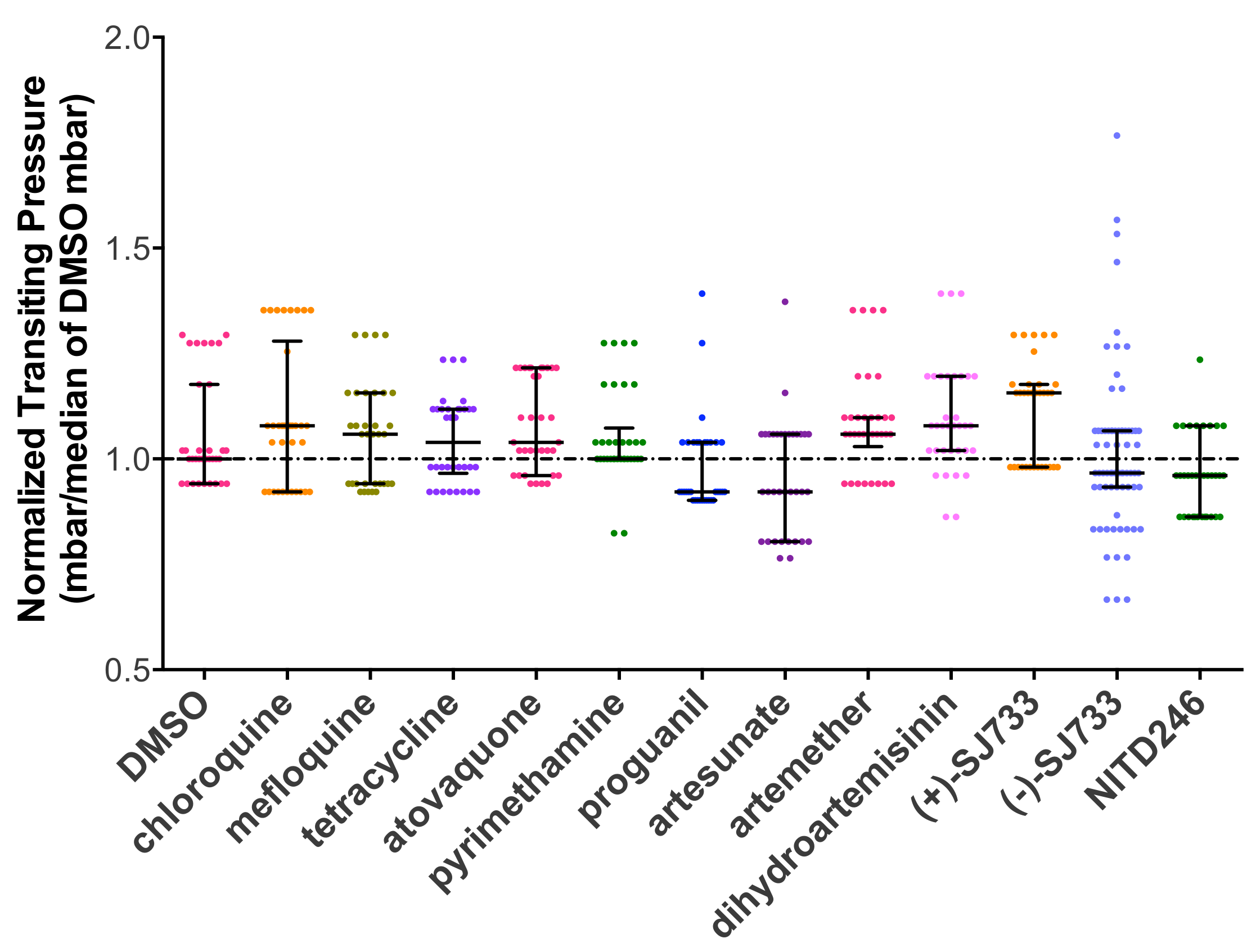

Supplement: Supplementary file 2 — 10.1186/s12936-015-0957-z Biophysical response of uninfected red blood cells to anti-malarial compounds. The anti-malarial compounds tested in this study did not significantly affect the deformability of uninfected RBCs. [file 12936_2015_957_MOESM2_ESM.tif]

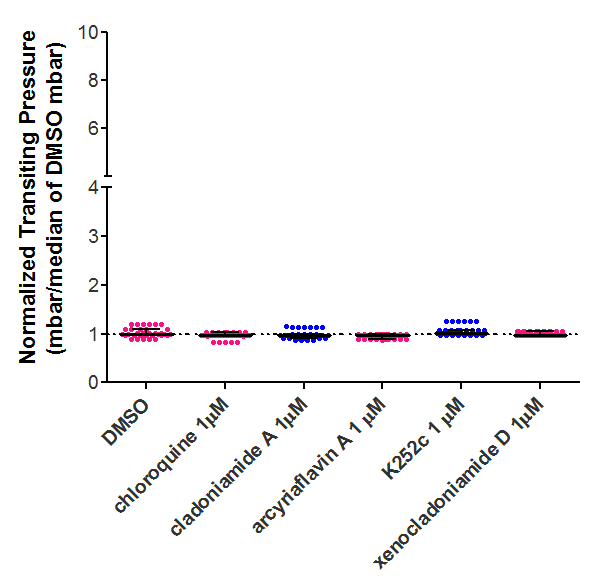

Supplement: Supplementary file 3 — 10.1186/s12936-015-0957-z Biophysical response of uninfected red blood cells to bisindole alkaloid compounds. Bisindole alkaloids tested did not significantly affect the deformability of uninfected RBCs. [file 12936_2015_957_MOESM3_ESM.tif]
